# Reduction of MRONJ risk after exodontia by virtue of ozone infiltration: A randomized clinical trial

**DOI:** 10.1111/odi.15006

**Published:** 2024-05-29

**Authors:** Olga Di Fede, Gaetano La Mantia, Carmine Del Gaizo, Rodolfo Mauceri, Domenica Matranga, Giuseppina Campisi

**Affiliations:** ^1^ Department of Precision Medicine in Medical Surgical and Critical Care (Me.Pre.C.C.), University of Palermo Palermo Italy; ^2^ Unit of Oral Medicine and Dentistry for Fragile Patients, Department of Rehabilitation, Fragility and Continuity of Care University Hospital Palermo Palermo Italy; ^3^ Department of Biomedical and Dental Sciences and Morphofunctional Imaging University of Messina Messina Italy; ^4^ Private Practice Palermo Italy; ^5^ Department of Health Promotion Sciences and Mother‐Child Care “G. D'Alessandro” University of Palermo Palermo Italy

**Keywords:** exodontia, healing, imaging, MRONJ, osteonecrosis of the jaw, ozone, prevention, tooth extraction

## Abstract

**Introduction:**

Exodontia is commonly considered as a risk factor for the development of medication‐related osteonecrosis of the jaw (MRONJ) in individuals exposed to bone modifying agents. This study was aimed at assessing the efficiency and safety of a gaseous oxygen–ozone mixture as an adjuvant to a standard exodontia to reduce the risk of MRONJ development.

**Methods:**

A randomized, open‐label, phase II, single‐center clinical trial involving 117 patients at risk of MRONJ was conducted. The study protocol tested injections of an oxygen–ozone mixture in the post‐extraction site. Participants were randomly assigned to two groups: oxygen‐ozone therapy, and standard tooth extraction protocol. Post‐extraction wound healing was assessed using the Inflammatory Proliferative Remodeling (IPR) Wound Healing Scale.

**Results:**

The oxygen‐ozone therapy group exhibited a significant improvement in wound healing post‐extraction during the inflammatory and proliferative phases, as indicated by the IPR scale scores at 3–5 days (*p* = 0.006) and 14 days (*p* < 0.001) respectively.

**Conclusion:**

Oxygen–ozone therapy shows promise in improving post‐extraction healing in patients at risk of MRONJ. Future studies with larger sample sizes and multicenter collaborations are recommended to confirm the validity of these findings and explore the long‐term efficacy of ozone therapy.

## 1 INTRODUCTION

Medication‐related osteonecrosis of the jaw (MRONJ) is a potentially severe adverse reaction, which can be defined as “the progressive destruction and necrosis of the mandibular and/or maxillary bone in patients who have received drugs with an established increased risk of the disease and who have not received prior radiation treatment” (Bedogni et al., 2023). MRONJ is primarily associated with the use of bone‐modifying agents (BMAs), specifically, with antiresorptive and/or antiangiogenic activities, which are administered to cancer and osteometabolic patients (Campisi et al., 2020). Furthermore, MRONJ can be characterized by symptoms such as: pain, discomfort, halitosis, and functional impairment (Campisi et al., 2020). MRONJ has traditionally been associated with dental extraction and other invasive dental procedures in patients receiving antiresorptive therapy (Liu et al., 2023). However, extensive and recent research has revealed that dental procedures may uncover the presence of already necrotic bone: these procedures could detect MRONJ without being the triggering event (Marcianò et al., 2021). Bone infection has been confirmed as one of the most important causative factors of MRONJ, rather than the dental surgical procedure itself (Otto et al., 2021). This is supported by the fact that preventive dental measures significantly reduce the incidence of MRONJ (Khan et al., 2015; Soutome et al., 2021; Yoneda et al., 2017). Soutome et al. (2021) have demonstrated that, in patients with teeth which are susceptible to infection, dental extraction can reduce the risk of developing MRONJ, even after the initiation of BMA therapy. Other risk factors worth mentioning are *cumulative dose*, *duration of antiresorptive therapy* and the *potency of the antiresorptive agent* (Coropciuc et al., 2023). Considering that infection is regarded as the most significant risk factor in the development of MRONJ, it is advisable to implement all necessary measures before, during, and after dental procedures to mitigate the risk of infection and subsequent development of MRONJ (Bedogni et al., 2024). This is also the case given that surgical and perioperative procedures have been suggested to mitigate this risk in patients undergoing dental extractions and bearing in mind patients' quality of life (Campisi et al., 2020; Kaibuchi et al., 2021). Prophylaxis by systemic and /or local antibiotics and antimicrobial mouthwashes are commonly recommended for dental extraction; healthcare providers should prioritize measures which minimize trauma to the surrounding tissues, thereby promoting optimal healing (Khan et al., 2015; Ruggiero et al., 2014). To minimize the risk of the development of MRONJ, it would be prudent to explore additional local and/or systemic treatment such as neo‐adjuvants or adjuvant therapy; thus, the healing process may be improved, particularly regarding comfort of the extraction site. To date, various supplementary treatments have been discussed in the literature, as described in Table 1.

**TABLE 1:** Various supplementary treatments to improve the healing process after tooth extraction.

	Therapy	Description
Systemic	Hyperbaric oxygen therapy	Involving the administration of high levels of oxygen in a pressurized environment (Ceponis et al., 2017; Freiberger et al., 2012; Watanabe et al., 2021)
	Teriparatide	A medication commonly used for treating osteoporosis, also demonstrating positive effects on bone healing and regeneration (Khan et al., 2016; Kwon & Kim, 2016; Sim et al., 2020)
Topical	Platelet‐rich plasma (PRP)	From the concentrated platelets of the patient's own blood (Di Fede et al., 2021; Mauceri et al., 2018)
	Low‐level laser therapy	Adjunctive therapy, which stimulates the cellular processes involved in tissue repair and regeneration (Di Fede et al., 2021; Mauceri et al., 2018)
	Mesenchymal stem cells (MSCs)	A promising treatment for a range of medical conditions, including post‐extraction healing. MSCs can differentiate into various cell types and promote tissue regeneration, thereby rendering them a preferred option for improving the healing process, following an extraction procedure (Li et al., 2022; Nifosì et al., 2021)
	Ozone therapy	Can be widely applied in the field of dentistry, each technique targeting specific oral and dental conditions with therapeutic benefits

### 1.1 Rationale

Managing dental extractions may prevent MRONJ, and both involve several important practices, as outlined by research and expert consensus. One pivotal recommendation is the adoption of minimally invasive surgical techniques to reduce bone trauma during dental extractions (Campisi et al., 2020; Coropciuc et al., 2023). Additionally, several studies and expert consensus support the use of perioperative antibiotic prophylaxis to prevent MRONJ in patients undergoing dental extractions (Bermúdez‐Bejarano et al., 2017; Poxleitner et al., 2017). The utilization of antibiotic prophylaxis as a preventive measure in this scenario is strongly recommended by the existing literature and expert recommendations (Campisi et al., 2020). However, the absence of standardized and clear protocols regarding antibiotic management remains a significant issue within the healthcare domain. This issue concerns: guidance relating to the drug type to administer, administration timing, dosage, quantity, and administration method (Ferlito et al., 2011; Ristow et al., 2021; Scoletta et al., 2011). On the one hand, it is crucial to minimize the risk of developing antibiotic resistance; on the other hand, it is essential to limit the duration of antibiotic therapy, thereby avoiding prolonged administration of antibiotics, which could compromise the systemic health of already vulnerable patients. A more structured and uniform approach at the protocol level would facilitate effective management of antibiotic treatment, simultaneously promoting treatment efficiency and preserving the long‐term effectiveness of antibiotics (Azher & Patel, 2021; Bermúdez‐Bejarano et al., 2017; Ferlito et al., 2011; Lodi et al., 2010; Matsumoto et al., 2017; Ristow et al., 2021; Saia et al., 2010; Scoletta et al., 2011).

Thus, in addressing this gap, this study proposes the use of ozonized gas as an adjuvant, in addition to antibiotic prophylaxis, during dental extraction procedures in patients at risk of MRONJ. This approach encompasses the aim of enhancing the current preventive measures and improving the overall patient outcomes.

Ozone can be applied as ozonated water, ozone gas insufflation, and as a gel containing ozonated oil (Sen & Sen, 2020). In the literature it has been suggested as a potentially effective alternative or adjunct to the two main therapeutic approaches currently used to treat MRONJ: antibiotic therapy and surgical intervention (Di Fede et al., 2022; Donati, 2019; Goker et al., 2020). However and with the exception of preventing dry socket in the extraction of third molars in patients not at risk of MRONJ (Materni et al., 2023; Sivalingam et al., 2017), there exist to date no recommendations for the preventive application of ozone as a perioperative procedure during dental extraction to reduce the risk of developing MRONJ. Patients can use ozonated water as an oral rinse in dental procedures, thereby effectively disinfecting the oral cavity (Arita et al., 2005; Sen & Sen, 2020). Oral gel containing ozonated oil has been demonstrated to be effective in promoting the healing of oral lesions, such as aphthous ulcers and cheilitis, as well as displaying beneficial effects in the treatment of oral candidiasis and denture‐induced stomatitis (Kshitish & Laxman, 2010; Sen & Sen, 2020).

Given the limited knowledge regarding peri‐operative ozone application and its potential to reduce the risk of developing MRONJ in patients undergoing dental extraction, the authors of this research urge directing future research in this direction. Thus, the aim of this study was to conduct a randomized, open‐label, single‐center, phase II clinical trial, with which to ascertain the efficiency and safety of oxygen–ozone mixture by infiltrations in dental extractions of patients at risk of MRONJ.

## 2 MATERIALS AND METHODS

The Consolidated Standards of Reporting Trials (CONSORT) checklist was used as a guideline for conducting and reporting this trial (Schulz et al., 2010). The study was open to all eligible patients according to the exclusion criteria, with no differences in gender or ethnicity. Ethical approval was obtained by the Ethics Committee of the Paolo Giaccone Policlinico University Hospital of Palermo (Approval Number 01/2018).

### 2.1 Trial design

The following was conducted: a randomized, open‐label, single‐center, and controlled clinical study, whose trial was registered with ClinicalTrials.gov (Identifier: NCT05036837). The study protocol was in accordance with the ethical guidelines of the 1964 Declaration of Helsinki and its later amendments or comparable ethical standards. All participants gave their written informed consent and the study was reported as a per Consort Statement (Schulz et al., 2010).

### 2.2 Participants

Potential participants were identified among patients attending the “Oral Medicine with Dentistry for Fragile Patients Unit” at the Paolo Giaccone Policlinico University Hospital of Palermo from February 2018 to March 2020. Patients were approached with written and verbal information about the study. Those who subsequently expressed interest in the study underwent screening, which was based on the eligibility criteria provided below:

#### 2.2.1 Inclusion criteria of patients

- Age ≥ 18 years
- At risk of MRONJ due to previous or current administration of drugs related to MRONJ
- Requiring dental extraction due to poor prognosis (severe caries and/or periodontitis)

#### 2.2.2 Exclusion criteria of patients

- Previous radiation in the head‐and‐neck area
- Neoplastic involvement of the jaw
- Previous diagnosis of MRONJ

Based on the above‐mentioned inclusion and exclusion criteria, patients were furnished with detailed information regarding the procedures. Subsequently, they voluntarily signed an informed consent form to participate in the study, receiving information regarding: the study, potential risks, and alternative treatments when possible.

#### 2.2.3 Settings and location data

Patients were treated at the “Oral Medicine with Dentistry for Fragile Patients Unit” (Paolo Giaccone Policlinico University Hospital, Palermo). Eligible patients were allocated different treatment types via a randomization program (REDCap) (Uschner et al., 2018), being assigned in a 1:2 ratio to receive either OZOPROMaF (Test group) or PROMaF (Control group) (Azienda Ospedaliera Universitario Policlinico “Paolo Giaccone” di Palermo Prevenzione e Ricerca Sull'Osteonecrosi Delle Ossa Mascellari Da Farmaci, n.d.).

### 2.3 Study intervention

In order to assess oral health status, an inspection and panoramic‐X ray were performed: if decay or periodontitis were suspected, a dental x‐ray was performed to eventually confirm poor prognosis prior to tooth extraction. Only when indicated (i.e., teeth in the vicinity of the inferior alveolar nerve and paranasal sinuses), was a cone beam computed tomography scan (CBCT) of the maxillofacial region performed. If MRONJ during tooth extraction was suspected, a biopsy of the post‐extraction alveolus was conducted. All dental extractions in the study were performed by the same expert operator (MR). Adjuvant therapy (intra‐mucosal perialveolar injections of an oxygen–ozone mixture) was carried out by two specialists (DFO and CDG), within the specialized “Oral Medicine with Dentistry for Fragile Patients” unit, adhering to uniform and standardized protocols. Beginning from the day prior to tooth extraction, the patient commenced medical treatment, which consisted of a combination of two different systemic antibiotics [Amoxicillin‐clavulanate 1 g (in case of penicillin allergy, erythromycin 600 mg) plus Metronidazole 500 mg], three times daily; local antiseptics (0.2% chlorhexidine mouthwash) were also prescribed. On the day of the surgery (T0), the surgical protocol involved: superficial local anesthesia (EMLA® cream; Astra, Westborough, MA), loco‐regional anesthesia, incision and flap debridement, tooth extraction, and osteoplasty.

On completion of the surgery:

- Control group: only the suture was positioned to maximize the approximation of wound margins.
- Test group: intra‐mucosal perialveolar injections of an oxygen–ozone (O_2_O_3_) mixture (15‐mL with a 26Gx 1/2–0.45 × 13 mm needle) and insufflation of the same mixture in the post‐extraction site for at least 1 min and hemostasis were performed, and the suture was positioned. Each patient was also scheduled for supplementary visits, during which the following were applied: 10 mL of the O_2_O_3_ mixture with a 26Gx 1/2–0.45 × 13 mm needle and insufflation of the same mixture in the post‐extraction site for at least 1 min, followed by hemostasis, and suture at T1 (3–5 days), T2 (14 days) and T3 (6 weeks) after the extraction.

### 2.4 Clinical outcome and measures

#### 2.4.1 Primary outcomes

The primary outcome of the study was to assess the healing response using the Inflammatory Proliferative Remodeling (IPR) wound healing scale at 6 weeks post‐surgery. The assessment of wound healing was conducted via the total score of the IPR scale, which provides a comprehensive assessment divided into distinct subscales; each subscale ranges from 0 to 1, resulting in a total score from 0 to 16 (Table 2). These subscales allow for the evaluation of the inflammatory response, proliferative response, and remodeling process. At the end of the follow‐up period (6 weeks), the total IPR score was calculated, ranging from 0 to 16. Scores between 0 and 4 indicated *poor healing*, scores between 5 and 10 indicated *acceptable healing*, while scores between 11 and 16 suggested *excellent healing* (Yahya et al., 2021).

**TABLE 2:** IPR (Inflammatory Proliferative Remodeling) Scale (Yahya et al., 2021).

T/phase	Parameter	Score 0	Score 1	Total score
Inflammatory T: 3–5 days	Bleeding (spontaneously or on palpation)	Yes	No	/8
	Granulation tissue	Yes	No	
	Haematoma	Yes	No	
	Tissue color	Redder or whiter than opposite side tissue	Like the opposite side tissue	
	Incision margins	Incomplete flap closure/fibrin clot/partial necrosis/complete necrosis	Complete flap closure/fine fibrin line	
	Suppuration	Yes	No	
	Edema NRS (1–10)	NRS 6–10	NRS 1–5	
	Pain NRS (1–10)	NRS 6–10	NRS 1–5	
Proliferative T: 14 days	Re‐epithelialization	Partial	Complete	/5
	Tissue color	Redder or whiter than opposite side tissue	Like the opposite side tissue	
	Scar	Scar wider than 2 mm/contour irregularity	No scar/scar less wide than 2 mm/contour regularity	
	Suppuration	Yes	No	
	Pain NRS (1–10)	NRS 6–10	NRS 1–5	
Remodeling T: 6 weeks	Scar	Scar wider than 2 mm/contour irregularity	No scar/scar less wide than 2 mm/contour regularity	/3
	Tissue color	Redder or whiter than opposite side tissue	Like the opposite side tissue	
	Pain NRS (1–10)	NRS 6–10	NRS 1–5	
Total process				/16

#### 2.4.2 Secondary outcomes

The secondary outcomes of the study evaluated the healing process using the scores of the IPR subclasses during the intermediate follow‐up stages at 3–5 days (T1) in the inflammatory phase, and at 14 days (T2) in the proliferative phase in the T group. Additionally, they assessed pain intensity using the Numerical Rating Scale (NRS) (Yahya et al., 2021).

### 2.5 Sample size and randomization

The trial design enrolled 101 patients (34 in the test group and 67 in the control group). It was calculated that this sample size would provide a 90% power, with which to detect a standardized mean difference equal to 1 between the two arms on the IPR scale at the 0.05 (two‐sided) level of significance, assuming equal known variances. This sample size calculation assumed that the mean total wound healing score on the IPR Scale for the PROMaF was 14.43 ± 1.45, as identified by Yahya et al. (2020) in a prospective study on healthy adult patients undergoing the surgical extraction of a wisdom tooth at a tertiary medical center. Assuming a dropout rate and/or non‐evaluable subjects of approximately 15%, enrollment reached 117 subjects (38 iTest group and 79 in the Control group). The randomization was generated on the computer using simple randomization software (RANDOM.ORG ‐ List Randomizer, n.d.).

#### 2.5.1 Statistical analyses

The statistical analysis was performed using R Statistical Software (v4.1.2; R Core Team, n.d.). Categorical variables were expressed as counts and percentages, with quantitative variables being expressed as the mean (standard deviation) or as the median and an interquartile range (the 25th and 75th percentiles), in cases of skewed distributions. The Chi‐square or the Fisher's exact test was used to compare groups T and C for categorical variables. The Student's *t*‐test or the Wilcoxon–Mann–Whitney test was calculated to compare groups T and C for the total IPR score, as a primary aim, and IPR at T1 and T2 and NRS, as secondary aims. Statistically significant differences were assessed using two‐sided *p*‐values below 0.05 (Table 4). At multivariable analysis, logistic regression was used to model binary responses (IPR > Median vs. IPR ≤ Median), as related to demographic, pharmacological, systemic, and clinical covariates, which were significant at univariable analysis. A comparison was also drawn among patient categories within the test group, with the *p*‐value derived from the Kruskal–Wallis rank sum test (Table 5). The analysis was conducted as ITT.

## 3 RESULTS

**FIGURE 1:**
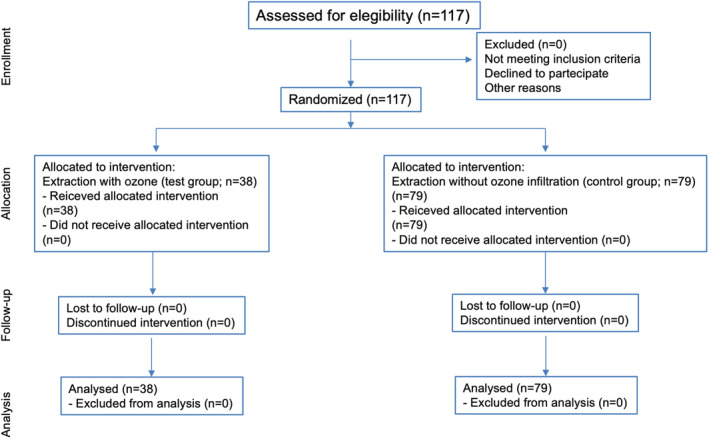
CONSORT flow diagram.

The study recruited 117 patients: 27 male and 90 female as shown in the CONSORT flow diagram (Figure 1). Of these patients, 54 had osteometabolic conditions, 57 were cancer patients, and 6 had both osteometabolic disease and cancer, categorizing them with cancer treatment‐induced bone loss (*CTIBL*). These clinical characteristics are presented in Table 3 below.

**TABLE 3:** Descriptive data of enrolled patients.

	Test group (T group) (*N* = 38)	Control group (C group) (*N* = 79)
Age		
Mean (SD)	66.7 (12.1)	69.6 (9.82)
Median [Q1, Q3]	71.00 [56.00, 74.75]	70.00 [63.00, 77.00]
Sex (%)		
F	27 (71.1)	63 (79.7)
M	11 (28.9)	16 (20.3)
Patient risk category (%)		
High	22 (57.9)	35 (44.3)
Low	16 (42.1)	44 (55.7)
Patient type (%)		
ONC (High‐dose BMA)	22 (57.9)	35 (44.3)
CTIBL (Low‐dose BMA)	4 (10.5)	2 (2.5)
OST (Low‐dose BMA)	12 (31.6)	42 (53.2)
Type of therapy (%)		
Antiresorptive medications	31 (81.6)	74 (93.7)
Anti‐angiogenic medications	2 (5.3)	1 (1.3)
Both	5 (13.2)	4 (5.1)

Medical and pharmacological data, including the dental history of patients, were recorded during the initial consultation. This included: age, sex, indications regarding the use of MRONJ‐related drugs, type and duration of MRONJ‐related medication use, medical history of chemotherapy, concurrent use of other medications, other concomitant diseases, and smoking habits. Patients who were administered BMA for variable periods were included in the study with no minimum or maximum exposure duration established.

The patients recruited for the study (Table 2) were categorized as: *ONC* (cancer patients undergoing high‐dose BMA treatment regarding oncological indications), *CTIBL* (Cancer treatment‐induced bone loss, Cancer patients undergoing low‐dose BMA therapies for the management of chemotherapy‐induced osteometabolic disorders), and *OST* (non‐oncologic patients taking low‐dose BMA for the treatment of osteometabolic conditions). Thus, two risk categories were identified, which were based on the drug type, administration route and cumulative dose: low and high risk. *ONC* patients were generally considered to be at high risk; *CTIBL* and *OST* patients were ascribed to the low‐risk category (Bedogni et al., 2023).

### 3.1 Primary objectives

A comprehensive overview of the results and comparative statistical analysis regarding the healing of post‐extraction sites between the test group and the control group is provided in Table 4.

**TABLE 4:** IPR comparative statistical analysis.

	T group (plus ozone therapy) (*N* = 38)	C group (controls) (*N* = 79)	*p*‐Value
Inflammatory T1 (3–5 days)^a^			
Mean (SD)	2.00 (0.870)	2.49 (0.875)	0.006
Median [Q1, Q3]	2.00 [1.00, 2.75]	2.00 [2.00, 3.00]	
Proliferative T2 (14 days)^a^			
Mean (SD)	4.24 (1.51)	3.70 (1.02)	<0.001
Median [Q1, Q3]	5.00 [4.00, 5.00]	4.00 [3.00, 4.00]	
Remodeling T3 (6 weeks)^a^			
Mean (SD)	3.00 (0)	2.96 (0.192)	0.230
Median [Q1, Q3]	3.00 [3.00, 3.00]	3.00 [3.00, 3.00]	
IPR_Total Process^a^			
Mean (SD)	9.37 (1.79)	9.15 (1.38)	0.275
Median [Q1, Q3]	9.50 [9.00, 10.00]	9.00 [8.00, 10.00]	

^a^ Wilcoxon signed‐rank test.

#### 3.1.1 Inflammatory phase

T group patients exhibited a mean score of 2.00, with a standard deviation of 0.870 during the inflammatory phase (T1–3–5 days after the intervention). In contrast, C group patients attained a mean score of 2.49 with a standard deviation of 0.875. Employing the Wilcoxon signed‐rank test, this statistical analysis revealed a notable difference between the groups, which was signified by a *p*‐value of 0.006. This indicated a significantly enhanced healing process regarding the T group during the critical phase of the inflammatory phase. Such an association was not confirmed at multivariable analysis.

#### 3.1.2 Proliferative phase

Passing to the proliferative phase (T2–14 days after the intervention), the results demonstrated that T group patients achieved a mean score of 4.24, with a standard deviation of 1.51; C group patients achieved a mean score of 3.70, accompanied by a standard deviation of 1.02. Here, the Wilcoxon signed‐rank test again manifested a highly significant discrepancy between the groups, which was characterized by a *p*‐value of <0.001. This emphasized a considerable improvement in healing within the ozone therapy group during this crucial phase. A lower probability of an elevated IPR at 14 days occurred with the C group than for the T group (AdjOR = 0.15, 95%CI = [0.06–0.38]) at multivariable analysis. During this phase, patients who were administered 2 weekly ozone infiltrations (out of a total of 9) displayed an improved recovery rate, with a score equal to or greater than 3.

#### 3.1.3 Remodeling phase

Shifting the focus to the remodeling phase (T3–6 weeks after the intervention), these outcomes confirmed that the T group patients group obtained a mean score of 3.00, in the absence of a standard deviation (SD = 0); C group patients achieved a mean score of 2.96, with a standard deviation of 0.192. In this latter instance, the Wilcoxon signed‐rank test did not reveal a statistically significant difference between the groups, with a *p*‐value of 0.230, thereby implying a relatively similar healing outcome between the two groups during this phase.

#### 3.1.4 IPR‐total process

Considering the total IPR process score, the data highlighted the fact that T group patients achieved a mean score of 9.37 with a standard deviation of 1.79, whereas C group patients attained a mean score of 9.15 and a standard deviation of 1.38. The Wilcoxon signed‐rank test again failed to demonstrate a statistically significant difference between the groups, yielding a *p*‐value of 0.275. This indicates a comparable overall evaluation of healing between patients who were treated with ozone and those who did not receive this treatment.

It is the opinion of the authors that the comprehensive statistical analysis outlined in this study has revealed the positive impact of ozone therapy on post‐extraction site healing, particularly during the inflammatory and proliferative phases. While no significant differences were observed during the remodeling phase and in the total IPR process score, it is contended that the results outlined in this research provide valuable insights into the potential benefits of ozone therapy in enhancing wound healing for patients at risk of MRONJ. Table 5 shows a detailed analysis of the healing of post‐extraction sites within the T group among different patient categories (ONC, OST, CTIBL), across various temporal phases (Inflammatory, Proliferative and Remodeling), and within the context of the overall IPR process.

**TABLE 5:** IPR comparison by patient category in the T group.

	ONC (*N* = 22)	OST (*N* = 12)	CTIBL (*N* = 4)	*p*‐Value^§^
Inflammatory T1 (3–5 days)				
Mean (SD)	1.86 (0.774)	2.25 (1.06)	2.00 (0.816)	0.612
Median [Min, Max]	2.00 [1.00, 3.00]	2.00 [1.00, 4.00]	2.00 [1.00, 3.00]	
Proliferative T2 (14 days)				
Mean (SD)	4.27 (1.55)	4.25 (1.42)	4.00 (2.00)	0.7991
Median [Min, Max]	5.00 [0, 5.00]	5.00 [0, 5.00]	5.00 [1.00, 5.00]	
Remodeling T3 (6 weeks)				
Mean (SD)	3.00 (0)	3.00 (0)	3.00 (0)	NA
Median [Min, Max]	3.00 [3.00, 3.00]	3.00 [3.00, 3.00]	3.00 [3.00, 3.00]	
IPR_total process				
Mean (SD)	9.36 (1.99)	9.50 (1.38)	9.00 (2.16)	0.925
Median [Min, Max]	9.50 [5.00, 15.0]	9.50 [6.00, 11.0]	9.50 [6.00, 11.0]	

^§^ *p*‐Value are from the Kruskal–Wallis rank sum test.

### 3.2 Secondary objectives

#### 3.2.1 Inflammatory phase in the T group

The mean values in the inflammatory phase revealed slight differences between the various categories: ONC displayed a mean value of 1.86, the OST value is 2.25, and 2.00 for CTIBL. However, the *p*‐value of 0.612 suggested that these differences were not statistically significant.

#### 3.2.2 Proliferative phase in the T group

The mean values in the proliferative phase were similar across the various categories: 4.27 for ONC, 4.25 for OST, and 4.00 for CTIBL. The *p*‐value of 0.7991 confirmed the absence of statistically significant differences between the patient categories.

#### 3.2.3 Remodeling phase in the T group

All groups exhibited the same mean of 3.00 in the remodeling phases but the standard deviation could not be calculated. This suggests a uniformity of results but an absence of variability prevented any calculation of statistical significance.

#### 3.2.4 IPR Total process in the T group

Mean values were comparable across the various categories in the overall IPR process: 9.36 for ONC, 9.50 for OST, and 9.00 for CTIBL. The *p*‐value of 0.925 indicated that the observed differences were not statistically significant. In conclusion, the analysis suggested that, in the various phases of post‐operative extraction site healing, there are no statistically significant differences among ONC, OST, and CTIBL patient categories. These findings are supported by the *p*‐values, which were obtained from the Kruskal–Wallis rank sum test.

#### 3.2.5 Pain assessment

In the assessment of pain utilizing the Numerical Rating Scale (NRS), no statistically significant differences were observed in pain levels and periods of healing duration between patients undergoing ozone therapy and those in the control group (*p* = 0.842) (Table 6).

**TABLE 6:** Pain assessment.

	Ozono terapy (*N* = 38)	Controls (*N* = 79)	*p*‐Value
Pain (NRS scale)			
Mean (SD)	3.71 (0.802)	3.70 (1.03)	0.842
Median [Q1, Q3]	4.00 [3.00, 4.00]	4.00 [3.00, 4.00]	

## 4 DISCUSSION

Several guidelines have been proposed in the scientific literature with which to manage dental extractions in patients at risk of Medication‐Related Osteonecrosis of the Jaw (MRONJ). Nevertheless, many of these studies present methodological limitations, including issues such as: inadequate randomization or control; insufficient sample sizes; and a wide range of diversified preventive approaches, such as antibiotic treatment, platelet‐rich plasma, and low‐level laser irradiation (Campisi et al., 2020; Kunchur et al., 2009; Tubiana‐Hulin et al., 2009). Prophylaxis using systemic antibiotics and/or local antimicrobial mouthwashes, concomitant with adequate alveoloplasty and primary wound closure, have been found to reduce the risk of MRONJ (Khan et al., 2015).

A 2007–2017 PubMed literature review regarding antibiotic prophylaxis and/or antibiotic therapy protocols for patients treated with bisphosphonates underlined the importance of antibiotic prophylaxis prior to oral surgical procedures in patients receiving low‐dose and high‐dose bisphosphonates in reducing the risk of MRONJ (Campisi et al., 2020). However, recommendations proposed in the literature regarding the timing of pre‐surgical antibiotic prescription vary: they range from one to a maximum of 7 days prior to extraction and continuing up to 30 days after dental extraction (Azher & Patel, 2021; Ferlito et al., 2011; Lodi et al., 2010; Matsumoto et al., 2017; Ristow et al., 2021; Saia et al., 2010; Scoletta et al., 2011).

Authors like Saia and, more recently, Capocasale and Varoni (Capocasale et al., 2021; Saia et al., 2010; Varoni et al., 2021) have provided indications demonstrating optimal results through the combined administration of two antibiotics, such as amoxicillin and metronidazole, for a limited period of time. In support of this, a recent review has emphasized the effectiveness of this combination as additional prophylaxis regarding the treatment plan for stage II‐III, grade C periodontitis (Karrabi & Baghani, 2022). Despite the etiopathogenetic differences between MRONJ and periodontitis, on the one hand, it is known that an infectious‐bacterial component is recognized in MRONJ; on the other hand, periodontitis is one of the local risk factors for the onset of MRONJ. To date, it has not been possible to determine whether these factors are consequential or concurrent (Goker et al., 2020).

Furthermore, it has been observed that the use of ozonized gas as an adjuvant (easy to administer, commercially available at a low cost, and devoid of adverse effects) has highlighted its efficacy in the post‐operative socket; this thereby significantly contributes to the clinical healing process of the extraction site (Chaudhry et al., 2021). This benefit is particularly evident during the initial stages of healing, especially during the inflammatory and proliferative periods, and particularly in patients at risk of developing MRONJ. Specifically, the data outlined in this paper have highlighted a comparison regarding the overall healing process score between Test group patients, who received ozone treatment, and Control group patients, who did not receive such treatment. The pain in all cases was manageable, and no local anesthesia was required in the days following tooth extraction in either of the patient groups. These results indicate that ozone treatment could positively impact the post‐extraction site healing, with a marked improvement during the inflammatory and proliferative phases. No significant differences emerged during the remodeling phase and in the total IPR score.

The wide‐sweeping action of ozone includes: virucidal and fungicidal properties, improved tissue oxygenation, wound healing support, oxygen metabolism balance, and immune system stimulation. Ozone has been employed in various studies relating to bone healing due to its immunomodulatory effects (Ripamonti et al., 2011). Indeed, the use of ozone reduces inflammation and pain and stimulates healing through the synthesis of interleukins, leukotrienes, and prostaglandins, all of which act as anti‐inflammatory agents (Adeyemo & Lagos, 2006). In addition to alleviating inflammation, ozone also stimulates the secretion of nitroglycerin, which acts as an arteriole vasodilator, thereby activating angiogenesis in inflamed tissue (Khan et al., 2010).

The findings outlined in this paper highlight the optimal healing of post‐extraction sites in 38 patients at risk of MRONJ, when supported by an extensive follow‐up period. Furthermore, it is hoped that the use of a standardized, clinical, assessment scale constitutes an innovative attempt to evaluate the post‐surgical healing trajectory in patients at risk of MRONJ, following dental extractions. It is important to note that the active intervention in this study involved the use of ozone therapy in combination with antibiotics, compared to standard surgery with antibiotics. The results emphasize not only the safety but also the effectiveness of ozone in the healing process of post‐extraction sites.

The Control group were treated with a standard procedure named PROMaF (protocol for extraction) (Bedogni et al., 2024; Di Fede et al., 2022). In detail, PROMaF (“Prevenzione e Ricerca Osteonecrosi delle ossa Mascellari da farmaci‐ the Prevention and Research of medication‐related Osteonecrosis of the jaw”) (Azienda Ospedaliera Universitario Policlinico “Paolo Giaccone” di Palermo Prevenzione e Ricerca Sull'Osteonecrosi Delle Ossa Mascellari Da Farmaci, n.d.). offers an eligible pathway for patients at risk of MRONJ at the Paolo Giaccone Policlinico University Hospital in Palermo.

This clinical assessment scale is based on the IPR Scale, which the authors of this research hope will prove to be appropriate in circumventing the limitations observed in previous wound healing assessment scales. The IPR Scale was meticulously devised to guide surgical decision‐making throughout the healing process; it is contingent upon a specific phase of treatment, be it inflammatory, proliferative, or remodeling (Hamzani & Chaushu, 2018). These constraints encompass: (1) the absence of a standardized definition of ideal wound healing; (2) challenges in distinguishing between distinct healing phases; and (3) the absence of any correlation between applied wound healing parameters and the various stages of surgical wound healing. Taking into consideration that the primary aim of this study invariably revolves around mitigating the risk of MRONJ during surgical procedures, this present study has various limitations: a sufficient but not large sample size; the single‐center nature of the investigation; and the heterogeneous history of systemic medications among subjects. Consequently, the endeavors of future research will be indispensable in confirming whether the utilization of ozone can indeed result in noticeable enhancements in clinical and osseous healing outcomes among patients at risk of MRONJ.

The authors of this study hope that the research described herein pioneers an innovative approach to treating MRONJ patients by virtue a prophylactic‐combined protocol for MRONJ, including medical/surgical procedures plus ozone infiltration. The compelling evidence emerging from this research supports the beneficial effects of ozone therapy on post‐extraction site healing.

This study underscores the need for further investigation into the prophylactic and therapeutic effects of infiltrative ozone therapy in patients at risk of MRONJ. The authors of this research remain optimistic that ongoing research will lead to enhanced treatment strategies, subsequently improving the well‐being and long‐term outcomes of MRONJ at‐risk patients. It is noteworthy that this study aspires to be pioneering in nature, being the first to employ the IPR scale for assessing various wound healing phases in the context of MRONJ. The authors of this paper contend that the utilization of the IPR scale provides a comprehensive framework for evaluating the efficacy of ozone therapy across different healing phases, further amplifying the significance of study findings. This innovative approach not only advances our understanding of MRONJ treatment, but it also underscores the importance of employing comprehensive assessment tools in future research within this domain. Despite the extensively documented analgesic properties of ozone, as evidenced by a vast body of scientific literature, the results documented in this study did not show statistically significant differences (Paoloni et al., 2009; Zhang et al., 2013; Zhuang et al., 2021). This intriguing aspect could be attributed to a multitude of complex variables which warrant careful consideration. The heterogeneity of the patients involved, with their diverse baseline conditions and individual responses to treatment, may have influenced the outcomes. Additionally, the specific modes of treatment administration and variations in clinical protocols might have had a significant impact on the results. It is also important to note the sample study size, which may not have been sufficiently representative to capture subtle differences or ensure adequate statistical power. Ultimately, these considerations underscore the importance of a comprehensive and balanced approach in interpreting research findings and in shaping future investigations, whose aim is to elucidate the effectiveness of ozone therapy in specific clinical contexts.

## 5 CONCLUSION

The application of oxygen–ozone therapy could present an innovative, powerful, and effective adjuvant for mitigating the risk of MRONJ and maximizing the efficacy of standard protocols (medical and surgery), particularly when operative procedures might pose an additional burden for complex cases and medical challenges. However, to consolidate and confirm the obtained results, it would be advisable to conduct further multicenter studies on a larger scale.

## AUTHOR CONTRIBUTIONS

**Olga Di Fede:** Conceptualization; methodology; validation; supervision; writing – review and editing; writing – original draft. **Gaetano La Mantia:** Investigation; validation; methodology; data curation; writing – original draft; resources. **Carmine Del Gaizo:** Data curation; validation; investigation; resources. **Rodolfo Mauceri:** Writing – original draft; conceptualization; methodology; supervision. **Domenica Matranga:** Investigation; validation; formal analysis; data curation; resources. **Giuseppina Campisi:** Resources; supervision; writing – review and editing; writing – original draft; conceptualization; validation; visualization; data curation.

## FUNDING INFORMATION

None.

## CONFLICT OF INTEREST STATEMENT

The authors declare that there are no conflicts of interest.

## INSTITUTIONAL REVIEW BOARD STATEMENT

The study was conducted in accordance with the Declaration of Helsinki and approved by the Ethics Committee of University Hospital of Palermo, Policlinico P. Giaccone (approval number 01/2018).

## INFORMED CONSENT STATEMENT

Informed consent was obtained from all subjects involved in the study. Written informed consent has been obtained from the patient(s) to publish this paper.

## Data Availability

The data that support the findings of this study are available from the corresponding author upon reasonable request.
